# Comparative genome analysis of microbial strains marketed for probiotic interventions: an extension of the Integrated Probiotic Database

**DOI:** 10.20517/mrr.2024.11

**Published:** 2024-09-03

**Authors:** Silvia Petraro, Chiara Tarracchini, Gabriele Andrea Lugli, Leonardo Mancabelli, Federico Fontana, Francesca Turroni, Marco Ventura, Christian Milani

**Affiliations:** ^1^Laboratory of Probiogenomics, Department of Chemistry, Life Sciences, and Environmental Sustainability University of Parma, Parma 43124, Italy.; ^2^Microbiome Research Hub, University of Parma, Parma 43124, Italy.; ^3^Department of Medicine and Surgery, University of Parma, Parma 43124, Italy.; ^#^Authors contributed equally.

**Keywords:** * Lactobacillus*, *Streptococcus thermophilus*, *Heyndrickxia coagulans*, *Bacillus*, genomics, probiotics

## Abstract

**Background:** Members of the *Bifidobacterium* genus and lactobacilli are the most commonly used probiotics to promote human health. In this context, genome-based *in silico* analyses have been demonstrated as a fast and reliable tool for identifying and characterizing health-promoting activities imputed to probiotics.

**Methods:** This study is an extension of the Integrated Probiotic Database (IPDB) previously created on probiotics of the genus *Bifidobacterium*, facilitating a comprehensive understanding of the genetic characteristics that contribute to the diverse spectrum of beneficial effects of probiotics. The strains integrated into this new version of the IPDB, such as various lactobacilli and strains belonging to the species *Streptococcus thermophilus* (*S. thermophilus*) and *Heyndrickxia coagulans* (*H. coagulans*) (formerly *Bacillus coagulans*), were selected based on the labels of probiotic formulations currently on the market and using the bacterial strains whose genome had already been sequenced. On these bacterial strains, comparative genome analyses were performed, mainly focusing on genetic factors that confer structural, functional, and chemical characteristics predicted to be involved in microbe-host and microbe-microbe interactions.

**Results:** Our investigations revealed marked inter- and intra-species variations in the genetic makeup associated with the biosynthesis of external structures and bioactive metabolites putatively associated with microbe- and host-microbe interactions.

**Conclusion:** Although genetic differences need to be confirmed as functional or phenotypic differences before any probiotic intervention, we believe that considering these divergences will aid in improving effective and personalized probiotic-based interventions.

## INTRODUCTION

According to the Food and Agricultural Organization of the United Nations (FAO) and the World Health Organization (WHO), probiotics are “live microorganisms which, when administered in adequate amounts, confer a health benefit on the host”^[1]^. The probiotic efficacy of specific bacteria mainly resides in their ability to enhance immune responses, metabolize nutrients generating beneficial metabolites such as short-chain fatty acids (SCFAs) and vitamins, and exert antagonistic interactions with pathogenic and opportunistic bacteria^[2-7]^. This is achieved by producing diverse organic acids, leading to a decrease in pH and the generation of hydrogen peroxide, lysozyme, and bacteriocins^[6]^. However, each strain used within dietary supplements must adhere to some basic minimum criteria, including safety for the intended use, the ability to interact with intestinal epithelial surfaces and mucous membranes, and an adequate tolerance to gastric and bile acids to survive in the human gastrointestinal tract^[8,9]^.

Generally, probiotic products may contain one or more microbial strains and are often developed with members of *Bifidobacterium*, *Bacillus*, and *Streptococcus* genera or with lactobacilli^[10,11]^. In particular, lactobacilli have been reported as the most prominent probiotic from the lactic acid bacteria (LAB) group*,* being widely used industrially due to their possible use in the production of dairy products such as cheese, yogurt, and other fermented milk-based foods^[12]^. The existing body of research links several lactobacilli, such as *Lactobacillus acidophilus* (*L. acidophilus*), *Lactobacillus bulgaricus* (*L. bulgaricus*), *Lacticaseibacillus casei* (*L. casei*), *Lacticaseibacillus rhamnosus* (*L. rhamnosus*), and *Limosilactobacillus reuteri* (*L. reuteri*), to the restoration of homeostasis in human intestinal disorders, inhibition of pathogen adhesion and growth, and a protective role against inflammatory diseases^[13-15]^.To date, despite the presence in the literature of comparative genomic studies on bacteria in relation to their probiotic qualities, which have led to the identification of genes involved in key metabolic pathways and potential probiotic characteristics^[16]^, a gap persists in analyses of the individual genetic factors underlying the specific claimed probiotic characteristics. For this reason, in this study, we investigated and compared the genetic content of 18 microbial strains commonly utilized in probiotic formulations, focusing on the species mentioned above. The goal was to expand the information cataloged in the Integrated Probiotic DataBase (IPDB) established in a previous study^[17]^, creating a comprehensive public repository that connects strain-specific genetic traits with potential health-promoting activities on the host.

## MATERIALS AND METHODS

### Genome sequences of strains employed in commercial probiotic products

Through extensive scrutiny of the scientific literature, several microbial species used in the production of commercial dietary probiotic products were initially identified. Moreover, we collected information regarding commercially available probiotic products worldwide, and an initial selection was subsequently made based on the bacterial species for which specific strains are specified on the label of the probiotic products. Finally, we screened for the bacterial strains whose genomes were deposited online in the National Center for Biotechnology Information (NCBI) Genomes database. In detail, a total of 15 lactobacilli, two strains of *Streptococcus thermophilus* (*S. thermophilus*), and a single strain of the *Heyndrickxia coagulans* (*H. coagulans*) species were retrieved and submitted to genomics and comparative genomics analysis [Table 1]. In addition, as an extension of the previously built genome database, this work integrates data from genome comparative analysis of 34 *Bifidobacterium* strains generated in the context of a previous study^[18]^. Data integration was performed while maintaining methodological consistency with the original research to ensure comparability and robustness of the results.

**Table 1:** List and general characteristics of the bacterial genomes analyzed in this study

**Organism name**	**Strain name**	**Accession number**	**Genome status**	**Genome size (Mb)**	**GC content (%)**	**CDS**
*Lactobacillus acidophilus*	BIO6307	GCA_008868625.1	Contig	1.97	34.6	1,850
*Lactobacillus acidophilus*	LA1	GCA_002286215.1	Complete	1.99	34.7	1,860
*Lactobacillus acidophilus*	La-14	GCA_000389675.2	Complete	1.99	34.7	1,867
*Lactobacillus acidophilus*	La-5	GCA_024665555.1	Complete	1.99	34.7	1,859
*Lactobacillus acidophilus*	NCFM	GCA_000011985.1	Complete	1.99	34.7	1,875
*Lacticaseibacillus casei*	BIO5773	GCA_008868595.1	Contig	3.08	47.9	2,848
*Lactobacillus gasseri*	BIO6369	GCA_008868535.1	Contig	1.87	35.1	1,807
*Lacticaseibacillus paracasei*	Lpc-37	GCA_002762235.1	Contig	3.16	46.3	3,010
*Limosilactobacillus reuteri*	RC-14	GCA_002762415.1	Contig	2.01	38.6	1,966
*Lacticaseibacillus rhamnosus*	ATCC53103	GCA_000026505.1	Complete	3.01	46.7	2,817
*Lacticaseibacillus rhamnosus*	BIO6870	GCA_008831425.1	Complete	3.01	46.7	2,815
*Lacticaseibacillus rhamnosus*	GG	GCA_003353455.1	Complete	3.01	46.7	2,819
*Lacticaseibacillus rhamnosus*	GR-1	GCA_900604925.1	Contig	2.89	46.5	2,682
*Lacticaseibacillus rhamnosus*	HN001	GCA_0001732155.2	Contig	2.91	46.74	2,689
*Ligilactobacillus salivarius*	BIO6313	GCA_013249205.1	Contig	2	32.6	1,931
*Streptococcus thermophilus*	TH-4	GCA_024665315.1	Complete	1.86	39.09	1,954
*Streptococcus thermophilus*	St-6	GCA_002796655.1	Contig	1.92	38.7	2,030
*Heyndrickxia coagulans*	Unique IS-2	GCA_01578455.1	Contig	3.45	46.4	3,332

CDS: Coding sequence.

### Genome annotation

To ensure consistent genomic analyses, open reading frames (ORFs) from the bacterial strains considered in this study were predicted and annotated using the most recent release of the MEGAnnotator pipeline^[19]^. In detail, contigs greater than 1000 bp were used to predict protein-encoding ORFs through Prodigal v2.0^[20]^. Functional annotation of the predicted ORFs was achieved using RAPSearch2^[21]^ (cutoff e-value of 1 × 10^-5^ and minimum alignment length 20) employing the NCBI reference sequences (RefSeq) database together with hidden Markov model profile (HMM) searches (http://hmmer.org/) against the manually curated Pfam-A database (cutoff e-value of 1 × 10^-10^). Subsequently, tRNA genes were detected through tRNAscan-SE v1.4^[22]^, and rRNA genes were identified using RNAmmer v1.2^[23]^.

### Comparative genome analyses

A pan-genomic analysis was performed using the Pangenome Analysis Pipeline (PGAP)^[22]^ on the 34 strains of the genus *Bifidobacterium* already present in the IPDB from a previous study^[18]^ and the 15 lactobacilli, the two strains of *S. thermophilus*, and the single strain of *H. coagulans* integrated into this new version of the IPDB [Supplementary Table 1]. Specifically, the proteome associated with each of these strains was classified through the gene family (GF) method into functional gene clusters by performing a pairwise protein similarity search using the software BLAST v2.2.28+ software (value and cutoff of 1 × 10^-10^ and exhibiting at least 50% identity on at least 80% of both protein sequences). Thus, the obtained protein data were used to assign the proteins to clusters of orthologous groups (COGs) using the Markov clustering algorithm based on graph theory (MCL)^[24]^. In this way, and with the use of a presence/absence matrix comprising the set of COGs identified at the level of the analyzed genomes (Linux command line “./PGAP.pl--strains [input_strain_list]--input input_path/--output output_path/--thread 20--identity 0.5--coverage 0.8--grappolo--method GF--evolution--pangenome”), the pan-genomic profile was reconstructed. The protein families shared among all the genomes were identified as part of the core genome, while protein families present in singleton were identified as Truly Unique Genes (TUGs).

Average pairwise nucleotide identity was calculated using the fastANI software^[25]^.

### Phylogenomic analysis

To study the phylogenetic relationships within the lactobacilli and the *Streptococcus* and the *Heyndrickxia* genera, the protein sequences shared among the members of the same genus were aligned using MAFFT software^[26]^. With ClustalW v2.1, using the neighbor-joining method, and with iTOL v6.8.1 (interactive Tree Of Life), an online tool for viewing, annotating, and managing phylogenetic trees, it was possible to obtain a graphic representation of the phylogenetic trees.

### Glycobiome prediction and identification of genes potentially involved in probiotic traits

The collected genomic sequences of the 15 lactobacilli and of two strains of *S. thermophilus*, as well as that corresponding to *H. coagulans* UNIQUE IS-2, were analyzed to evaluate the possible presence of genes involved in persistence and survival in the gastrointestinal tract and microbial metabolic activity with potential roles in promoting the host’s health. Specifically, screening of genetic traits involved in the production of pili, S-layers, adhesins, and teichoic acids was performed through BLASTp sequence similarity searches^[27]^ (cutoff e-value of 1 × 10^-10^) exploiting *ad hoc* databases built with well-known protein sequences retrieved from NCBI database, as previously performed^[17]^. The designation “hypothetical protein” refers to genes whose specific function has not yet been fully characterized. However, using screening approaches based on homology searches with known functional databases, it was possible to identify structural domains within these hypothetical proteins that suggest their potential function. The location of gene clusters encoding exopolysaccharides (EPS) structure was predicted based on homology with known sequences encoding EPS. The glycosyltransferase priming gene, crucial for forming such structures, was manually verified to distinguish these clusters from other glycan clusters. In addition, the absence of genes characteristic of other glycan clusters was assessed. BAGEL 4.0^[28]^ and AntiSMASH 5.0^[29]^ were used to predict antimicrobial compounds, while the Cazy database (December 2023 update) was exploited to identify glycosyl hydrolase families^[30]^. Genes encoding the enzymes responsible for the last step in the SCFA, and vitamin biosynthetic pathways were retrieved from the MetaCyc database^[31]^, and their presence in the collected probiotic genomes was evaluated through local BLASTp searches. All the results were then manually validated to eliminate possible false positives.

## RESULTS AND DISCUSSION

### Retrieval of genomic sequences corresponding with probiotics

In a previous study^[17]^, a genome database named the IPDB was built, including the genome sequences of *Bifidobacterium* genus members used in commercial probiotic products. As an extension of the IPDB, we have enriched the database with the chromosome sequences of several lactobacilli as well as those of strains belonging to the genus *Heyndrickxia* (previously belonging to the *Bacillus* genus) and *Streptococcus* members currently used in bacteria-based supplements available in the global probiotic market.

Specifically, we performed exhaustive research on probiotic product labels to identify those strains used in bacteria-based products currently available worldwide, obtaining 70 different strain IDs belonging to the species listed above [Supplementary Table 2]. However, out of these, only the different lactobacilli selected for the study, the two strains of *S. thermophilus* species, and one strain of *H. coagulans* [formerly known as *Bacillus coagulans* or *Weizmannia coagulans*]^[32-35]^ were suitable for the subsequent analyses as their associated genomes (complete or drafts) were available in public repositories such as the NCBI genome database [Supplementary Table 2]. Accordingly, although numerous studies have analyzed the genetic makeup of different probiotics to identify genetic factors related to their probiotic characteristics, there is still a need for more detailed and integrated analyses that can comprehensively elucidate the genetic basis underlying the health benefits these bacteria can confer. Indeed, access to essential genetic information is vital for capturing the mechanisms underlying the interactions between probiotics and the human host and identifying potential risks associated with specific strains.

The 18 genome sequences included in the study were *de novo* annotated and integrated into the IPDB. The complete list of the bacterial strains and their general genomic features are reported in Table 1. This updated version of the IPDB, encompassing genome sequences as well as manually curated information of strain-specific functional particulars, is freely accessible at http://probiogenomics.unipr.it/cmu/ (direct download at http://probiogenomics.unipr.it/files/IPDB_latest.zip).

### Phylogenomic and comparative genomic analyses of bacterial chromosomes included in the IPDB

The genomes of 18 commercially distributed probiotic strains were employed to perform comparative genomic analyses to identify intra- and inter-species genetic peculiarities. The Average Nucleotide Identity (ANI) analysis performed upon alignment of lactobacilli genomes used in probiotic supplements highlighted a higher degree of genome identity among the five strains of *L. acidophilus* (average of 99.98%) as well as among three (ATCC53103, GG, and BIO6870) of the five strains belonging to the *L. rhamnosus* species (mean of 99.99%) [Supplementary Table 3]. These results suggest that not only *L. acidophilus* strains, known for their genome identity among commercial isolates^[36]^, but also identical strains of *L. rhamnosus* could have been deposited and commercialized as probiotics with different labels. This observation may limit comparisons between strains and necessitates a detailed taxonomic review to clarify strain identities.

To provide a comprehensive perspective on the genetic diversity of various lactobacilli and of the 34 strains of *Bifidobacterium* already included in the previous version of the IPDB, the two strains of *S. thermophilus* and the strain of *H. coagulans* included in this new version of the IPDB compared to others, we performed species-level pangenome and phylogenomic analyses incorporating additional complete genome sequences available in public databases and pertaining to the same probiotic species selected for this study [Supplementary Table 1]. This approach enabled us to compare many conspecific genome sequences to identify actual genetic variations between these.

The pangenomic analysis highlighted a variable number of TUGs, ranging from 12 to 403, corresponding to *Bifidobacterium animalis* subsp. *lactis* SD5219 and *B. longum* subsp. *infantis* EVC001, respectively [Supplementary Table 4]. However, within the lactobacilli, *Ligilactobacillus salivarius* (*L. salivarius*) BIO6313 and *L. reuteri* RC-14 were the bacterial strains with the largest TUGs (175 and 186, respectively). In contrast, *L. acidophilus* is similar to what was previously reported for *Bifidobacterium animalis* subsp. *lactis*^[36]^ is confirmed to be an isogenic species characterized by extremely low diversity and a high degree of genomic conservation.

**Figure 1:**
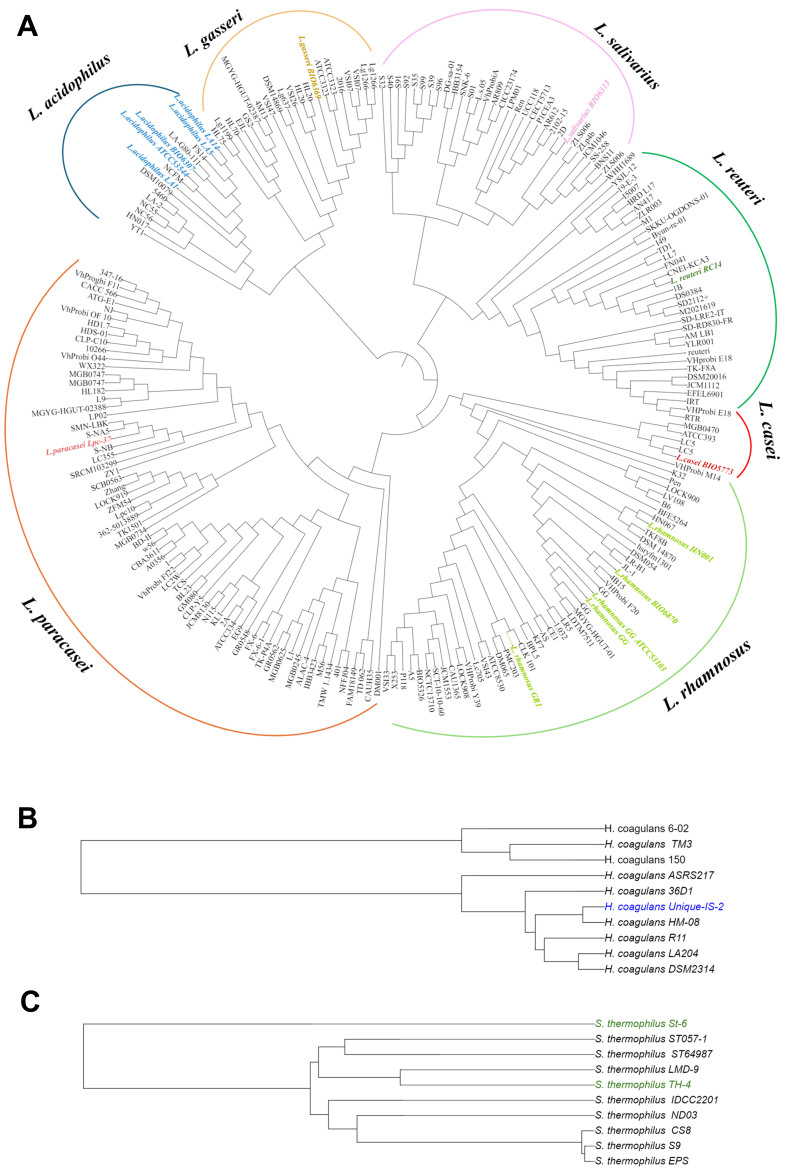
Phylogenomic trees of the bacterial genera added in this study to the IPDB. Panels (A-C) show the phylogenomic tree of the lactobacilli and of *Heyndrickxia coagulans* and *Streptococcus thermophilus* genus, respectively. All trees were constructed based on species-specific core genomes derived from a comparison of strains used in commercial probiotic formulations (highlighted in colored text) along with additional publicly available complete genome sequences of the same species. IPDB: Integrated Probiotic Database.

In accordance with the relatively low number of identified strain-specific genetic traits, our phylogenomic analysis based on the core genome revealed that the genomes of lactobacilli and the *H. coagulans* strain used in probiotic supplements are uniformly placed within their respective species-specific phylogenetic clusters [Figure 1A and B]. Conversely, the genome of *S. thermophilus* St-6, harboring 316 TUGs [Supplementary Table 4], is placed on a distinct branch within the phylogenomic tree of its species [Figure 1C]. Although some caution is needed when interpreting phylogenomic trees reconstructed with both completed and draft bacterial chromosomes, from our analysis, it seems plausible that the probiotic strain *S. thermophilus* St-6, unlike *S. thermophilus* TH-4, may harbor genetic traits that distinguish it from other conspecific genomes [Figure 1C]. The detailed analysis of the specific differences between the strains is an excellent starting point for future studies, as it may provide new insights into the unique characteristics of the various probiotic strains and their application potential.

### Genes involved in persistence in the gastrointestinal tract

The distribution of various genetic traits related to the ability to persist in the gastrointestinal tract and to induce beneficial effects in the host was investigated among the selected probiotic strains of lactobacilli and the *S. thermophilus* and *H. coagulans* genus, as well as among members of *Bifidobacterium* genus already included in the IPDB.

Specifically, the ability to adhere, at least temporarily, to the host’s gastrointestinal mucosa is a highly desired feature for probiotic strains, allowing for optimal adaptation to and persistence in the new niches encountered in the host^[37]^. Specific bacterial extracellular structures are recognized for their pivotal role in gut colonization, biofilm formation, and modulation of host immune system responses^[38]^. For these reasons, in this study, we explored the arsenal of genes encoding pili, S-layer proteins, mucus-binding adhesins, teichoic acids, and exopolysaccharides within the genome sequences of the selected probiotic strains.

#### Variability in pili-encoding gene sequences

Bacterial pili are filamentous appendages extending from the cell surface, exerting pivotal functions in interaction and adhesion to host cells, other bacteria, or environmental surfaces^[39,40]^. Since previous *in vitro* studies have led to the identification of pili in bacterial strains^[41]^, we analyzed the genomes of the 18 probiotic strains included in this study to assess the presence of gene sequences encoding for pili. Our genome-wide explorations revealed that all the bacterial genomes considered possibly produced external adhesion proteins. In particular, lactobacilli generally encode cell wall anchoring proteins that harbor LPXTG motifs, which are already known among LAB as motifs that can enhance the properties and duration of bacterial colonization in the gastrointestinal tract and their probiotic effects on the host^[42]^ [ Supplementary Table 5]. At the same time, only closely related members of the *L. casei* and *Lacticaseibacillus paracasei* (*L. paracasei*) species, along with *L. rhamnosus* GG and BIO6870 strains, demonstrated the presence of two distinct pili sortase-dependent encoding islands known as *SpaFED* and *SpaCBA* pilus operons known for their involvement in intestinal mucus adhesion^[43]^ [ Supplementary Table 5]. In contrast, other examined *L. rhamnosus* strains (GR1 and HN001) appeared to possess only the *SpaFED* locus [Supplementary Table 5]. In accordance with previous observations^[44,45]^, transposon genes are flanking the *SpaCBA* cluster in *L. rhamnosus* GG but are absent in the *L. casei* BIO5773 genome, suggesting that *SpaCBA* in *L. rhamnosus* GG might be acquired through horizontal gene transfer (HGT) events.

It is worth mentioning that strain-dependent differences in the pili-encoding gene clusters were also observed within members of the *Bifidobacterium* genus previously included in the IPDB^[17]^, showing a noticeable divergence of genetic potential for sortase-dependent (SD) pili and a high level of genetic conservation of the *Tight Adherence* pili encoding locus (*Tad locus*).

#### Genetic potential for mucus-binding adhesins and S-layer proteins

Similar to the pili structures described above**,** adhesins and S-layer proteins on the bacterium’s outer surface facilitate interactions between the bacteria and the external environment. They probably play a role in adhesion to various substrates, mucins, and host cells and in processes involving intimate interaction with other bacteria^[46,47]^. Based on our in-silico investigation, the lactobacilli analyzed were predicted to encode a wide range of host-specific adhesion proteins, from one to 11, depending on the species [Supplementary Table 6]. Specifically, most lactobacilli appeared to express multiple adhesin types interacting with mucin (Mub domain), fibronectin (Fbp domain), or fibrinogen protein, with *L. acidophilus* and *L. gasseri* showing the greatest genetic potential to produce proteinaceous host-specific adhesion factors [Supplementary Table 6]. The specific binding abilities of these extracellular proteins, particularly Mub proteins, and FbpA-B from *L. acidophilus*, have been proposed to play crucial roles in facilitating effective colonization and competitively displacing gut pathogens^[48]^. In contrast, among the screened bacterial genomes, S-layer-associated proteins (SLPs and SLAPs) were precisely identified only in those from *L. acidophilus* species, underlying differences in possible mucus- and microbe-microbe interactions which could significantly affect the probiotic potential of different lactobacilli [Supplementary Table 7]. Indeed, previous studies have shown that S-layer proteins are essential for the probiotic effect of certain lactobacilli, as they enable colonization of the intestine and are responsible for their immunomodulatory properties^[45]^, as also confirmed *in vitro* studies^[49]^. Furthermore, through homology analysis between these sequences, we could rule out that the sequences belonged to the same strain, given the low percentage of identity found (average homology of 42%).

In parallel, the same analysis performed on the two strains of *S. thermophilus* included in the extended version of the IPDB database showed that they lacked coding sequences for both S-layer and pili proteins as well as genetic determinants for adhesion on mucin, thus casting doubts on their ability to competitively colonize and persist in the human gut [Supplementary Tables 5-7], as previously demonstrated^[50]^.

#### Repertoire of lipoteichoic acids and exopolysaccharide biosynthesis gene clusters

**Figure 2:**
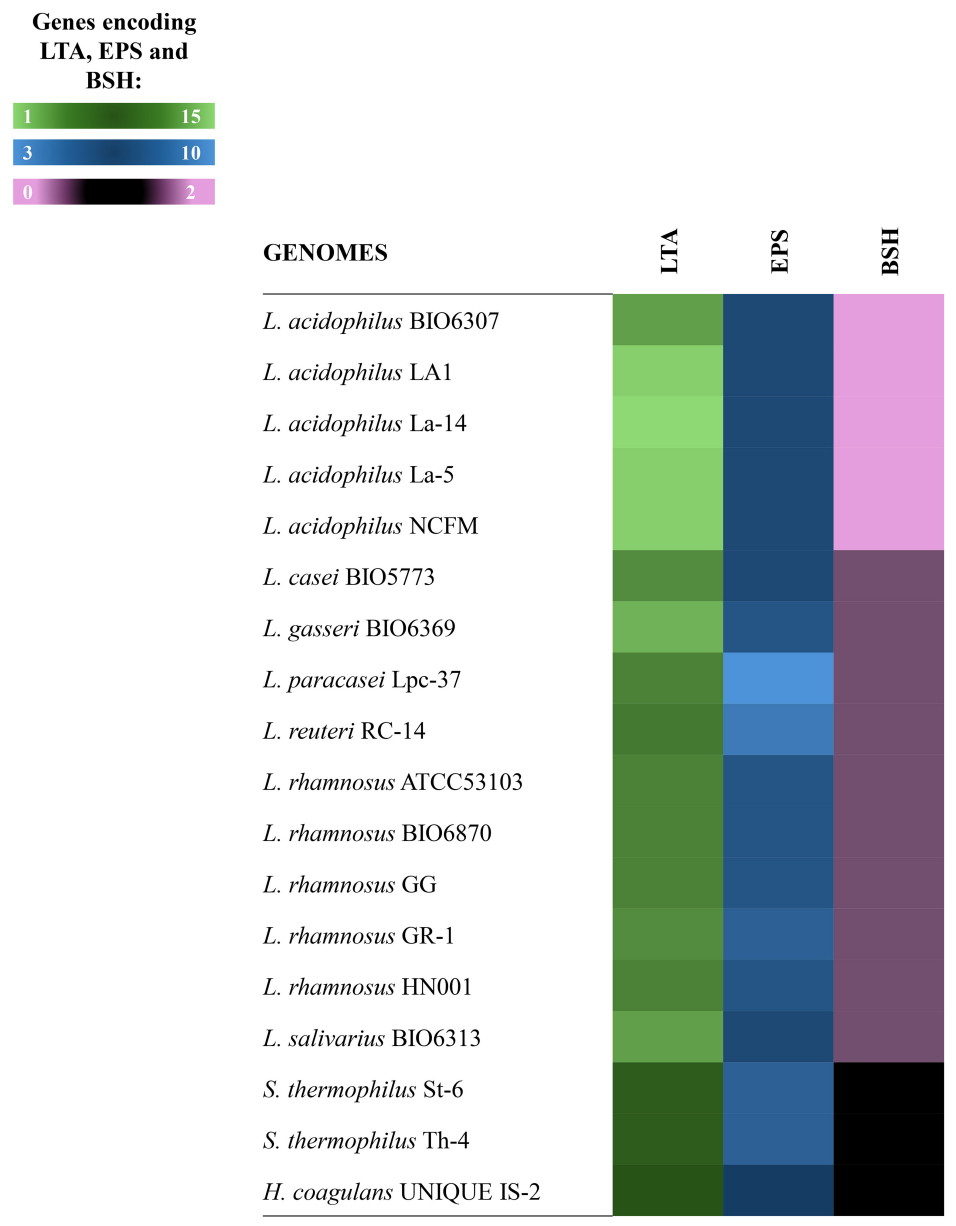
Investigation of the microbial genetic traits involved in LTA, EPS, and BSH biosynthesis. The heat map illustrates the genome-level presence (colored box) or absence (black box) of genes involved in the biosynthesis of LTA (green), EPS (blue), and BSH (pink). The color scale reflects the number of significant sequences found for each function analyzed: black indicates a complete absence of gene function in the probiotic under investigation, darker colors indicate a small number of significant sequences identified (with a value of 1 indicating minimal presence), and lighter colors indicate a larger number of significant sequences identified for the gene function analyzed. LTA: Lipoteichoic acid; EPS: exopolysaccharides; BSH: bile salt hydrolase.

In Gram-positive bacteria, the cell wall is decorated with additional surface components, including lipoteichoic acids (LTAs) and EPS, exerting crucial roles in bacteria-host crosstalk by mediating immune response and anti-inflammatory functions^[51-53]^. Therefore, information at the genome level concerning such capsular polysaccharides derived from probiotics is important. These investigations showed that all probiotic strains included in the IPDB possessed putative LTAs-related genes coding for LTA synthase, LTA-transporters, and D- alanylation protein [Figure 2 and Supplementary Table 8].

The increased number of putative LTA gene islands in *L. acidophilus* members, particularly in LA1, La-14, La-5, and NCFM strains, could contribute to their anti-inflammatory effects and immunomodulatory benefits^[54,55]^. However, although the contribution of lactobacilli LTAs in modulating the intestinal innate immune response has been demonstrated^[56,57]^, more in-depth genomic examination of these genetic determinants is needed to better understand their interaction with the immune system.

In contrast, the *S. thermophilus* strains and the *H. coagulans* strain showed fewer gene sequences coding for LTA genome-wide. This result might suggest that these bacteria might have a different ability to interact with the host immune system than *L. acidophilus* strains.

The genome-wide analysis was then extended by evaluating the possible presence of sequences coding for EPS [Figure 2, Supplementary Table 9], the presence of which in LAB has already been evidenced by *in vitro* studies^[58,59]^. Beyond being present in all bifidobacterial strains previously investigated^[17]^, putative gene clusters encoding EPS were found in all analyzed lactobacilli genomes, as well as *S. thermophilus* and *H. coagulans* species, confirming the extensive history of knowledge regarding the high EPS production by these bacteria commonly employed in probiotic formulations^[60-62]^. Notably, previous studies^[55]^ have established that EPSs are present in LAB and highlighted their significant impact on immune cells. They can activate macrophages and stimulate lymphocyte proliferation. Therefore, the presence of EPS in bacterial cells is a crucial factor to consider when analyzing these microorganisms’ probiotic potential. However, a marked variation in the organization of the genes in the EPS clusters was observed among the considered bacterial chromosomes. This result is consistent with previous studies reporting high variability of EPS clusters between different lactobacilli genomes and different *Streptococcus* genomes^[63,64]^. Previous studies showed the existence of six different EPS genotypes in *S. thermophilus* strains^[65]^ and a highly variable genetic composition in EPS-encoding gene clusters depending on the habitat of origin of the lactobacilli strains considered^[63,64]^. In particular, a high degree of conservation emerged for gene sequences directly involved in EPS biosynthesis, such as epsA, epsB, epsC, epsD, and epsE, while the products genes supporting this process by encoding enzymes that transfer I sugar monomers, ensuring passage through the membrane and polymerization, showed high variability in the number and composition of protein families.

#### Bile salt hydrolase encoding genes

Another essential bacterial trait needed to survive across the host gastrointestinal tract is the ability to detoxify bile salts through specific chemical modifications^[66]^. This function is entrusted to the gene product of bile salt hydrolase (BSH), the presence of which has been verified in several bacterial genera used as probiotics, including in the genomes of lactobacilli^[67]^ and has long been recognized as a selection criterion for defining potential probiotics. The BSH enzyme plays a pivotal role in bile acid metabolism and enables bacteria expressing it to benefit host health by positively influencing the intestinal metabolome, improving fat digestion, increasing the bioavailability of fat-soluble nutrients, and providing benefits on lipid metabolism^[67]^. Screening for *BSH* genes within the bacterial chromosomes included in the IPDB showed that 100% of lactobacilli and bifidobacteria possessed such coding sequences, which, on the contrary, was absent in *S. thermophilus* and *H. coagulans* genomes [Figure 2, Supplementary Table 10]. Nevertheless, it is noteworthy to mention that the unique composition of spores morphology can provide *H. coagulans* members with high tolerance to acidic conditions in the stomach upon ingestion^[68]^.

Overall, results from our genome-based investigations highlighted a high inter-strains variability in the genetic potential for microbe- and host-microbe interactions and, consequently, in their expected performance to adhere and persist in the host’s gastrointestinal tract as well as in the stimulation of the immune system. Accordingly, these results highlighted the need for genomic-based approaches to understand and predict the functionality of probiotics at the strain level.

### Genes involved in microbial functionalities with health-promoting roles

Along external structures involved in mechanical interactions with the surrounding environment, bacteria are capable of producing various primary and secondary metabolites, including glycosidic hydrolases (GHs) and bacteriocins, that can greatly influence the host metabolism as well as microbe-microbe relationships^[69-72]^.

#### Genetic potential for bacteriocins production

Lactic acid bacteria can produce protein compounds known as bacteriocins, which have been shown to have bactericidal or bacteriostatic effects on closely related bacteria or others, including pathogenic bacteria. In particular, previous studies have shown that bacteriocins can inhibit the adhesion of pathogenic microorganisms in the host due to their antimicrobial action. In detail, they can induce pore formation in the cell wall of the target organism or inhibit cell wall biosynthesis, causing its death^[73]^. For this reason, bacteriocin production is generally regarded as a desirable probiotic trait, and numerous bacteriocins have been isolated over time, mainly from lactobacilli^[74-76]^. Based on our screening, bacteriocin and bacteriocin-related coding sequences were present in the genome of all microbial strains analyzed in this study, with a putative higher bacteriocinogenic potential in lactobacilli, particularly those from *L. acidophilus* and *L. rhamnosus* species [Supplementary Table 11]. These divergences across probiotics suggested an unequal distribution of bacteriocin production potential, likely leading to varied abilities to compete and establish within a complex community, such as the human gut microbiota.

#### Differences in carbohydrate metabolism

To establish and proliferate in the host gut environment, bacteria exploit a great variety of GHs to metabolize complex carbohydrates and derive nutrients necessary for their growth, exerting simultaneously beneficial effects on gastrointestinal homeostasis^[77]^. In this regard, understanding the repertoire of genes involved in carbohydrate metabolism, i.e., the glycobiome, within probiotic strains is of paramount importance to gain insights into the metabolic pathways that contribute to the health-promoting activities of bacteria, enabling targeted and strategic employment of the therapeutic potential of these microorganisms^[77]^.

**Figure 3:**
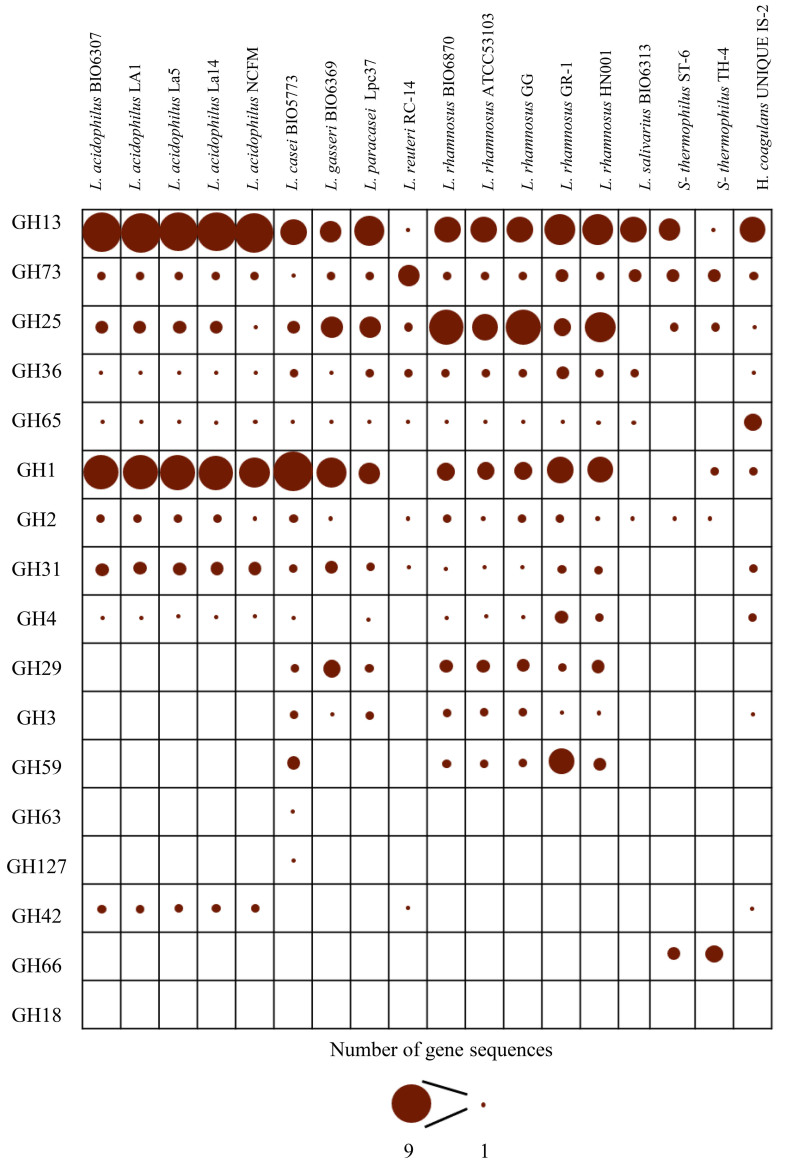
Glycobiome analysis of the genome sequences newly included in the IPDB. The bubble chart reports the number of gene sequences corresponding to various GHs identified within the genome sequences examined in this study. For each investigated genomes, the size of the circles is proportional to the number of genes corresponding to a given GH. IPDB: Integrated Probiotic Database; GHs: glycosidic hydrolases.

Compared with the probiotic *Bifidobacterium* strains previously included in the IPDB, which were enriched in gene sequences encoding GHs responsible for the degradation of breastmilk polysaccharides and intestinal mucins, lactobacilli, and *S. thermophilus,* and *H. coagulans* species have exhibited a preferential commitment to complex plant-derived polysaccharides. Specifically, among the analyzed genomes, the most widely distributed glycoside hydrolases were GH13 and GH73 [Figure 3, Supplementary Tables 12 and 13], while the highest number of sequences were recorded for GH13 and GH1 (114 and 88, respectively) [Figure 3, Supplementary Tables 12 and 13], whose members, as noted on the public CAZy database^[78]^, are mainly active on substrates containing α-glucoside linkages, such as starch, and involved in converting cellulose into glucose, respectively^[79,80]^.

However, glycobiome analysis also showed marked interspecies differences with species-associated peculiarities [Figure 3, Supplementary Tables 12 and 13]. For example, *L. acidophilus* strains were characterized by family GH42 coding sequences, whose members are primarily beta-galactosidases metabolizing lactose to galactose and glucose^[3]^.

#### Genes involved in organic acids production

**Figure 4:**
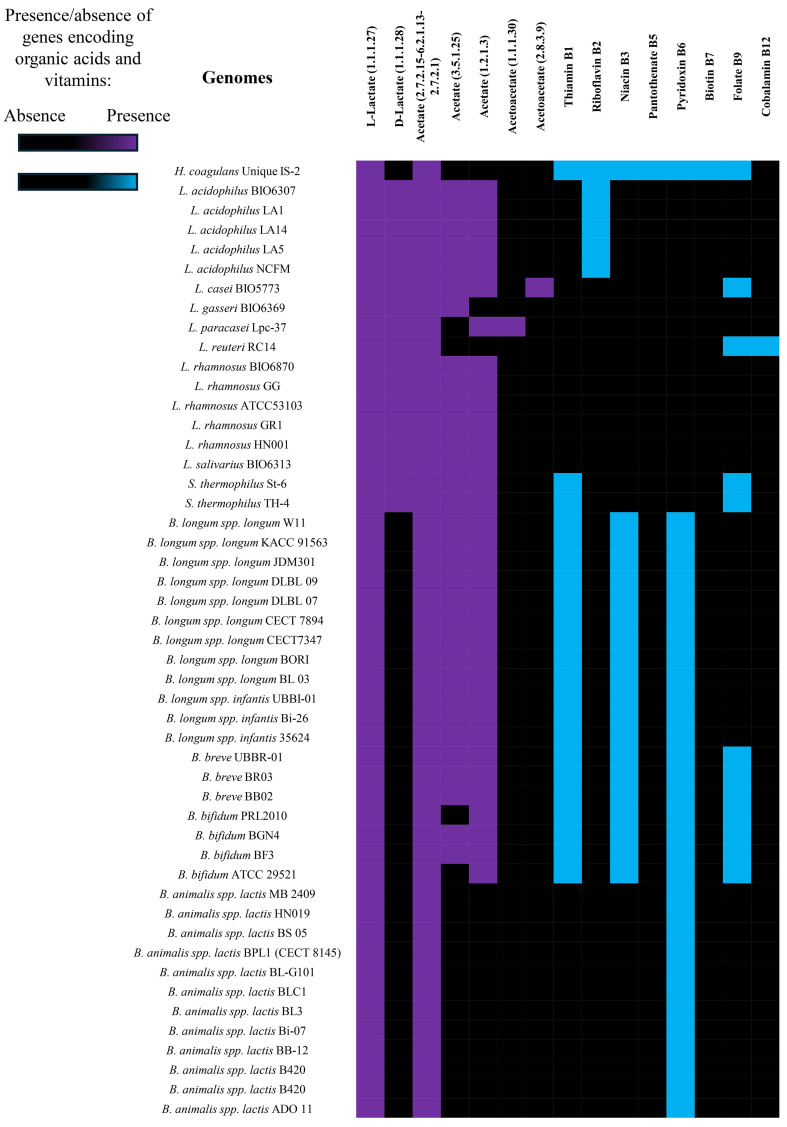
Investigation of the microbial genetic traits involved in SCFAs and vitamin biosynthesis. The heat map illustrates the genome-level presence (colored box) or absence (black box) of genes involved in the biosynthesis of organic acids (violet) and B-group vitamins (light blue). These investigations are based on the search for the gene encoding the final enzyme in the pathway, which is specified through the EC number reported on the first row. SCFAs: Short-chain fatty acids; EC: Enzyme Commission.

As a result of bacterial carbohydrate metabolism, lactobacilli and bifidobacteria produce organic acids, mainly acetate and lactate^[81,82]^. These metabolites have been associated with positive effects on intestinal homeostasis, immune response, and inhibition of potential pathogens due to the decrease in pH^[83-85]^. Moreover, it has been proposed that acetate could activate lactobacilli bacteriocin biosynthesis^[86]^. According to the well-characterized key synthesis genes, all lactobacilli and *Bifidobacterium* genera members, as well as *S. thermophilus* and *H. coagulans* UNIQUE IS-2 strains, appeared able to produce acetate and L-lactate, with evident inter and intra-species variability [Figure 4, Supplementary Table 13]. Only lactobacilli and *S. thermophilus* strains showed coding sequences for D-lactate [Figure 4, Supplementary Table 14]. Interestingly, gut commensal-derived D-lactate recently showed stronger immune regulation effects than L-lactate^[87,88]^, allowing us to suggest different abilities among probiotics to ameliorate gut dysbiosis and protect the host from enteropathogenic infections.

#### In-silico prediction of vitamin biosynthesis

Certain bacteria synthesize water-soluble vitamins during their microbial metabolism, such as those of the B group^[89,90]^. Accordingly, we assessed the presence of genes encoding enzymes involved in the final step of group B vitamin pathways in the genomes of lactobacilli, *S. thermophilus*, and *H. coagulans* UNIQUE IS-2*,* extending the analysis to the *Bifidobacterium* strains already included in the IPDB^[17]^. Our results showed marked intraspecies differences, with the *H. coagulans* UNIQUE IS-2 strain appearing as the strain with the most extensive genetic potential for vitamin biosynthesis [Figure 4, Supplementary Table 14]. In contrast, although certain strains of *L. acidophilus* have been reported as being able to increase folate in fermented milk^[89]^, the genomes of *L. acidophilus* strains collected in the IPDB lack the genes involved in folate biosynthesis [Figure 4, Supplementary Table 14], while the genome sequences of *Bifidobacterium* species contained the coding sequences for vitamin B1, B3, B6, and B9 [Figure 4, Supplementary Table 14].

Accordingly, the differences that emerged in bacterial vitamin biosynthetic potential may reflect strain-dependent metabolism that, thus, should be considered when designing multi-species probiotic formulations to optimize their beneficial impact on host health.

### Probiotic strains: analysis of antibiotic resistance and virulence factors for safety

When using a bacterial strain as a probiotic, it is paramount to assess its safety and risk/benefit ratio^[90]^. To date, three main factors are considered in selecting a strain as a probiotic, including the general aspects of the strain, including origin, identity, safety, non-pathogenicity, and resistance; technical elements, such as growth properties *in vitro* and during processing, ability to survive and remain viable during transport and storage; and functional aspects and its advantageous characteristics^[91]^. In particular, the safety and non-pathogenicity of a bacterial strain intended for probiotic use are considered factors of primary importance^[91]^. Consequently, assessing the presence of genes that confer antibiotic resistance could support *in vivo* and *in vitro* investigations. In addition, such assessment is crucial to ensure consumer safety and prevent the spread of antibiotic resistance, which seriously threatens public health.

In this context, the 18 probiotic strains integrated into this new version of the IPDB were examined for possible determinants of antibiotic resistance and virulence factors [Supplementary Tables 15 and 16]. Although our *in silico* analyses are for predictive purposes only, this approach may provide a starting point for future *in vitro* validations. The results showed that the St-6 and TH-4 strains of *S. thermophilus* possessed genes presumably involved in resistance against several classes of antibiotics [Supplementary Table 15], including rifampin, aminocoumarin antibiotics, as well as penicillin and other beta-lactam antibiotics. In addition, only strain St-6 had a 23S rRNA methyltransferase, which acts on the dimethylation of 23S rRNA, conferring resistance to macrolides, lincosamides, and streptogramins B^[92,93]^ [Supplementary Table 15]. Performing the same analysis within lactobacilli, only strain RC-14 of *L. reuteri* was predicted to have a gene encoding for a Lincosamide nucleotidyltransferase (LNUA), which adenylates the hydroxyl group of lincosamides, modifying their structure and preventing their binding to their target [Supplementary Table 15].

Concerning virulence-associated factors, we identified two key genes that deserve attention for their potential role in modulating the host immune system. Specifically, strain BIO6313 of *L. salivarius* was predicted to have specific sequences (lgtC) belonging to the LOS system (VF0044), which is involved in the immune modulation of the host, in which it can activate strong inflammatory responses^[94]^. In addition, the two *S. thermophilus* strains were shown to possess a UTP-glucose-1-phosphate uridyl-transferase, which can catalyze UDP-glucose pyrophosphorylase production. Previous studies have emphasized this enzyme’s importance in hyaluronic acid capsule synthesis^[89]^, which protects bacteria from phagocytosis and promotes the evasion mechanisms of the host immune system.

These results suggest that *L. salivarius* BIO6313 and *S. thermophilus* strains (St-6 and TH-4) may possess virulence factors capable of modulating the host immune response [Supplementary Table 16]. Although these observations are based on *in silico* analyses and need further experimental validation, they may suggest that the probiotics tested are generally free of virulence and antibiotic-resistance genes.

### Analysis of genes associated with prebiotics and sugars of interest

The interaction between the gut microbiota and food components directly affects the health and well-being of host organisms^[95]^. Prebiotics and specific sugars are known to play a crucial role in modulating the composition and activity of the gut microbiota^[96,97]^. Some prebiotics such as fructo-oligosaccharides (FOS), xylo-oligosaccharides (XOS), manno-oligosaccharides (MOS), and raffinose are known for their ability to stimulate the growth of beneficial bacteria. In contrast, although not classified as prebiotics, lactose and glycogen still significantly impact host well-being. Lactose can be utilized by lactic acid bacteria as a source of energy and carbon via lactic fermentation, offering potential benefits for lactose-intolerant individuals who cannot independently metabolize this sugar due to deficiency of the enzyme β-galactosidase^[98]^. At the same time, precedent studies^[99]^ have shown that the ability of bacteria to metabolize glycogen contributes to their retention and probiotic activities in the gastrointestinal tract.

Genomic analysis of genes involved in metabolizing prebiotics and sugars is essential for understanding the functionality of gut bacteria. In the new IPDB, we evaluated the presence of these sequences in 15 strains of lactobacilli, two strains of *Streptococcus*, and the *Heyndrickxia* strain. Our findings showed that *L. gasseri* strain Lpc-37 contains specific gene sequences for a fructan beta-fructosidase [Supplementary Table 17]. This enzyme breaks down fructans into simpler fructose units, facilitating their absorption or fermentation by other intestinal bacteria, providing significant benefits in reducing symptoms associated with gastrointestinal disorders. In addition, strains BIO6870, GG, HN001, GR-1 of *L. rhamnosus*, strain ATCC53103 of *L. reuteri*, and strain BIO5773 of *L. acidophilus* have genes for xylanase. This enzyme degrades xylan to XOS, a potential prebiotic that stimulates microbial growth [Supplementary Table 18]. The *H. coagulans* Unique IS-2 strain possesses genes coding for enzymes that remove acetyl groups from polysaccharides, suggesting a mechanism for influencing the structure of complex dietary polysaccharides and modulating gut microbial ecology [Supplementary Table 17]. This enzymatic activity results in the release, in the gastrointestinal tract (GIT), of simpler substrates that could be used by other members of the gut microbial community as nutrient sources, thus promoting the growth of beneficial bacteria that are not directly capable of metabolizing complex polysaccharides and contributing to the development of a more balanced and diverse gut environment, promoting host health. It is, moreover, relevant to note that all strains of *L. rhamnosus*, Unique IS-2 strain of *H. coagulans*, *BIO6369* strain of *L. casei, RC-14* strain of *L. paracasei*, ATCC53103 strain of *L. reuteri*, Lpc-32 strain of *L. gasseri*, and BIO6307 and BIO5773 strains of *L. acidophilus* have genes for alpha-galactosidase [Supplementary Table 19]. This enzyme degrades raffinose, a complex sugar found in various foods, potentially improving the digestion of this sugar in the gut and reducing gastrointestinal disorders associated with its fermentation.

A detailed analysis of the metabolic capacity of the included probiotic strains revealed that all strains of *L. acidophilus*, strain St-6 of *S. thermophilus,* and strain TH-4 of *L. salivarius* possess gene sequences for enzymes that degrade lactose and glycogen [Supplementary Tables 20 and 21], complex sugars widely present in the human diet. In contrast, *L. rhamnosus* strains, *L. reuteri* strain ATCC53103, *L. gasseri* strain Lpc-37, and *H. coagulans* strain have genes for enzymes capable of degrading glycogen but not lactose [Supplementary Table 21].

### Conclusion

In this study, we retrieved 18 publicly available genome sequences of lactobacilli and *S. thermophilus,* and *H. coagulans* (formerly *Bacillus coagulans)* species currently used in many probiotic supplements that were used for genomics investigations to expand the IPDB previously build^[17]^. However, it is important to note that the number of sequences used is limited by the availability of data in public databases for probiotics used in commercial products, recognizing this as a significant constraint. As the genomes of bacterial species not yet included in the IPDB become available, they can be used for further studies, further expanding our understanding of the genomic properties of probiotics. By examining and comparing these strains*,* we recognized noteworthy differences in their genetic makeup with possible consequences on their health-promoting activities and mechanisms of action. Specifically, strain-dependent genetic potentials were observed for external adhesive proteins such as pili and S-layer components and metabolic activities, including carbohydrate metabolism and vitamin biosynthesis. Remarkably, such differences could impact the ability of bacteria to adhere to host surfaces and modulate metabolic processes, ultimately affecting their overall functional outcomes in promoting health.

Considering the rapid advances in research on the human gut microbiome and probiotic fields^[83,84]^, we believe that it is essential to account for genetic variations related to probiotic properties in the formulation of multi-species probiotics to amplify and optimize their positive effects on host health. To achieve this goal, extensive whole-genome sequencing is needed to improve our understanding of the genetic content of the strains used in probiotic products^[85]^. Indeed, through detailed knowledge of the different metabolic capabilities of bacterial strains, we can strategically tailor probiotic treatments and adapt probiotic formulations to meet specific host health needs more effectively. However, it is essential to emphasize that using a specific bacterial strain as a probiotic cannot be based solely on its genome content but on its impact on health, which must be demonstrated in well-designed and controlled interventional studies. Consequently, it is paramount to associate *in silico* studies with functional and phenotypic investigations before any probiotic intervention.

## DECLARATIONS

### Availability of data and materials

The IPDB can be accessed at http://probiogenomics.unipr.it/cmu/ (direct download at http://probiogenomics.unipr.it/files/IPDB_latest.zip).

### Copyright

© The Author(s) 2024.
